# How patient-physician encounters in critical medical situations affect trust: results of a national survey

**DOI:** 10.1186/1472-6963-4-24

**Published:** 2004-09-07

**Authors:** Rahul A Shenolikar, Rajesh Balkrishnan, Mark A Hall

**Affiliations:** 1 Division of Management, Policy and Community Health, University of Texas School of Public Health, 1200 Herman Pressler, Houston, TX 77030, USA; 2 Wake Forest University Schools of Law and Medicine, 2000 West First Street, Piedmont Plaza II, 2^nd ^Floor, Winston-Salem, NC 27104, USA, Winston-Salem, USA

## Abstract

**Background:**

Patients' trust in physicians and in the medical profession is vital for a successful patient-physician relationship. Trust is especially salient in critical medical situations, such as serious side-effects, hospitalizations, and diagnoses of serious medical conditions, but most trust studies have been done with the general population or in routine primary care settings. This study examines the association between patient-physician encounters in such critical medical situations and patients' trust in their physician and in the medical profession in general.

**Methods:**

A random national telephone survey was conducted using validated multi-item questionnaire measuring trust and satisfaction with physicians and with the medical profession. A seven item questionnaire measured the patient-physician encounters in critical medical situations. A total of 1117 subjects aged 20 years and older with health insurance were included for analyses. Spearman rank order correlations were used to determine the association of encounter variables with trust in physicians and the medical profession.

**Results:**

Prescription of medications by primary care physicians that patients believed might have side effects was negatively correlated with trust in physician (ρ = -0.12, p < 0.001, n = 1045) in multivariate analysis. A primary care physician evaluating the patient for a condition the patient believed was serious was positively correlated with trust in physician (ρ= 0.08, p < 0.01). Being hospitalized was positively correlated with trust in the medical profession (ρ = 0.12, p < 0.01, n = 475).

**Conclusion:**

Hospitalization, perceived seriousness of condition, and concerns about the risks of medications were found to be associated with patient trust in physicians or the medical profession. These findings highlight the salience of trust in serious physician-patient encounters and the role that patient vulnerability plays in determining patient trust.

## Background

Patients' trust in their physicians is vital for a successful treatment relationship [1,2], which is important for achieving desired treatment outcomes [3-6]. Trust in physicians is a positive acceptance of a vulnerable situation in which patients believe that physicians will care for their interests [7]. Previous studies have identified three sets of factors that are associated with trust: patient characteristics, physician characteristics, and relationship factors. [2,8-26] Numerous factors, such as choice of physician, length of relationship, and managed care settings have been found to be among the stronger predictors of trust.[16] However, previous studies have not examined the association between trust and the patient-physician encounters in critical medical situations. Experience of interactions in some critical medical situations is potentially a very important factor because it affects the salience of trust, and because of the role that vulnerability is thought to play in the psychology of trust. [7] Some examples of critical medical situations are those in which physicians diagnose serious medical conditions, perform surgery, or prescribe medication that might have serious side effects. The heightened vulnerability created by these situations could have pronounced effects, either positive or negative, on trust in one's physician or in the medical profession. The objective of this exploratory study is to examine the possible associations between patients' experiences in critical medical situations and their trust in their physician and in the medical profession in general.

## Methods

### Sample selection

The national sample was selected by random digit dialing, with the sampling frame generated by a random sample from a proprietary database of working residential telephone exchanges in the continental United States. The sampling frame was provided by Survey Sampling, Inc. of Westport, Connecticut. Survey Sampling, Inc. maintains a database of working residential telephone exchanges in the continental United States. In selecting the numbers to be called in this study, an exchange was randomly selected and then a random number between 0000 and 9999 was generated to complete the number. This process was repeated until a sufficient quantity of numbers had been generated. Between April-June 1999, a total of 4028 numbers dialed (minimum 15 attempts each) yielded 2637 (65 %) responses. Households were excluded with no one over the age of 20 (n = 66) or where the adult respondent with the next birthday did not have health insurance (n = 151) or had not seen a health professional at least twice during the past two years (n = 248). Respondent selection within eligible households was done using the next birthday method. Contacts with the 2172 potentially eligible individuals resulted in the following dispositions: 1117 (51.4%) were interviewed; 571 (26.3%) refused; 484 (22.2%) were unable to participate (not home, ill, non-English-speaking).

### Instrument

All subjects were asked a core set of questions about their regular health care provider, demographic characteristics, satisfaction with care, physical and mental health, and preferences regarding seeking care and making medical decisions. Satisfaction was measured in two ways: a single item on patients' satisfaction with their physicians and a 12-item scale on patients' satisfaction with the healthcare that they have been receiving from all sources during the past few years. Two validated trust scales were used [12,22], each using a 5-point Likert scale [a 10 item physician trust scale (Cronbach's α = 0.93), and an 11 item medical profession trust scale (Cronbach's α = 0.92)]. The physician trust scale asked mainly about trust in primary care physicians. Items in both the scales represent four dimensions of trust (fidelity, competence, honesty, global). Physician trust was measured by the sum of 10 items scores, ranging from 10 to 50, with a higher score indicating more trust. Trust in the medical profession was measured by 11 item scores ranging from 11 to 55, with a higher score indicating more trust. Patient satisfaction with health care was measured using a previously validated 12-item 60 point scale. [27] Other variables thought to be related to physicians trust were measured as follows: whether one had enough choice in selecting a physician (yes/no); number of years with physician; willingness to recommend to friends (strongly agree to strongly disagree; past disagreement or dispute with the physician (yes/no); desire to switch physicians (strongly to strongly disagree). Due to interview length (25 minutes), only half of the subjects that were randomly selected were asked about trust in the medical profession. [12]

A questionnaire naming seven medical situations with dichotomous responses (Yes/No) was developed to identify encounters with physicians separately for the patient's primary physicians and for other physicians if their services were utilized. The items in the questionnaire were created after expert review by a panel of physicians, behavioral scientists, and health lawyers and piloted in patient focus groups.[22]These items asked whether over the past five years, the subject had been hospitalized, had undergone minor (non-anesthesia) or major surgery, had been prescribed medications that they thought could have serious side effects, had been evaluated for possible or actual cancer or for another serious medical condition, or had been referred to a specialist.

### Analyses

Dependent variables for the study were physician trust and medical profession trust. Independent variables included patient-physician encounter variables and other significant variables mentioned in the previous section identified from a previous study. [5] These hypothesized predictors were tested for their bivariate association with trust scores using Spearman rank-order correlations. Finally, partial Spearman correlations between significant predictors in the bivariate analyses (adjusting for other confounders, e.g. we examined correlation between subjects who had undergone major surgery and physician trust adjusting for other variables such as their satisfaction with healthcare, poor physical health, number of visits to physician, whether their physician was a foreign physician, long waiting time with physician, and disputes with physician) and the corresponding measures of trust were estimated. All statistical analyses were conducted using STATA statistical software (College Station, TX). [28]

## Results

Table 1 presents descriptive statistics on the study population. One study population refers to the respondents who were asked about trust in physicians, and the second study population refers to respondents who responded to questions about trust in the medical profession. Complete data for analyses were obtained for 1045 subjects for the physician trust analysis, and 475 subjects for the analysis of trust in the medical profession. Mean patient characteristics, including the encounter variables, did not differ very much across the two study populations.

Table 2 presents results of the bivariate Spearman rank-order correlations of the encounter variables with corresponding measures of trust in primary care physician. Prescribing medications to patients that they thought would have serious side effects, overnight hospitalization, and evaluating patients for a serious condition were found to be significantly correlated to physician trust (all significant at p < 0.05) as well as trust in the medical profession (all significant at p < 0.05) while the physician performing a surgery (p < 0.05) perceived as major by the patient was significantly correlated to only trust in physicians. Significant predictors in the bivariate analysis were included in the multivariate partial correlation analysis.

Table 3 shows the partial Spearman rank order correlations between different correlates of trust, adjusting for each other. Patient-physician encounter variables significant in bivariate analyses were included. Physician trust was negatively correlated with prescription of a medication that a patient thought had serious side-effects (p < 0.01). An encounter which included being evaluated for a serious condition other than cancer was associated with higher trust in physicians (p < 0.01). Trust in the medical profession was significantly higher in patients who had been hospitalized by their primary care physician (p < 0.01). None of the other encounter variables were associated with profession trust. In other findings (detailed results not shown), we did not identify any physician-patient encounter variable that was significantly correlated with trust in the subject's primary physician among those who had an interaction with a physician other than their primary physician (33% of study population).

## Discussion

These findings suggest that the patient-physician encounters in critical medical situations are associated with patients' trust in physicians. Of seven conditions that indicate more serious experiences, four were found to have a significant relationship with either trust in one's primary physician or trust in the medical profession. The three conditions not found to be significantly related to trust include the two that were less threatening than the others – minor surgery, and referral to a specialist. The significance of critical medical situations might arise either from the salience of trust in these situations, or from the vulnerability that is created by more serious medical conditions or procedures.

It is important to note that the relationship with trust was positive for two intensity indicators, but negative for a third. The positive relationship lends support to the theory that trust arises from vulnerability and therefore is potentially higher when there is a greater need to trust. [7] However, vulnerability can also give rise to distrust. The negative correlation with the variable relating to medication might arise from the fact that the wording of this variable may have indicated that the physician made a mistake in prescribing the wrong medication. Other encounter variables were neutral regarding physician competence.

Another interesting pattern that emerged in this initial, exploratory analysis is the type of trust that related to different encounter variables. Most variables were related to trust in the subject's primary physician, but overnight hospitalization was also related to increased trust in the medical profession, and when other predictors of trust were controlled for, significance remained only for trust in the medical profession. This is consistent with the fact that hospital treatment is more of a team effort that reflects on the performance of the medical system. Other encounter variables that were significant for both types of trust in the bivariate analysis remained significant only for physician trust after adjusting for other predictors of trust. This is consistent with the theory that the primary object of trust in most treatment settings is the treating physician.

Due to the limitations of this study, these findings and interpretations should be regarded as preliminary. This was an exploratory cross-sectional study whose particular inclusion criteria resulted in a sample that does not exactly correspond to the socio-economic distribution of the general United States population. The cross-sectional design leaves open the possibility that current levels of trust could affect recall of past events. The study asked about the impact of previous encounters on current physician trust, but it should be noted that the current physician may not be the one involved in previous encounters. Another limitation is that it was not possible to know the degree of vulnerability associated with each type of experience. Also, most measures were based on subjects' unverified reports.

Summarizing the results, this study found significant associations between patient-physician encounters in medical situations and trust in primary physicians and in the medical profession. These associations reflect the role that vulnerability plays in the psychology of trust. Future research should focus on identifying in further detail which forms of encounters affect which types of trust, and in what directions. Also, more research is needed to understand why these encounters affect trust and what factors modify these relationships. Such research could help to identify threats to trust and further maintain trust and trustworthy conditions. This is especially important in the current era in which many people fear that trust in medical care is rapidly eroding.[16]

## Competing interests

None declared.

## Authors' contributions

RB and RAS conceived the paper. RB and MAH were responsible for data collection. RB and RAS conducted the data analyses. RAS was primarily responsible for writing the paper. The manuscript was reviewed and critically revised by RB and MAH.

## Pre-publication history

The pre-publication history for this paper can be accessed here:



## Figures and Tables

**Table 1:** Descriptive Statistics of Study Population

**Variable **⇓	Physician Trust Population*(n = 1045)		Medical profession Trust Population** (n = 475)	
Physician trust [10–50]	40.7 (6.1)		40.6 (6.1)		
Profession trust [11–55]			33.5 (6.9)	
Satisfaction with health care [12–60]	41.9 (7.6)		41.0 (7.3)		
Age [21–40 years]	34.1%		34.4%	
Age [41–64 years]	36.4%		41.2%	
Age [65 years and above]	29.5%		24.4%	
Male gender	33%		33%	
Latin origin	4%		5%	
Non white	24%		17%	
Low income (< $20,000/year)	17%		18%	
Poor physical health	13%		14%	
Adequate choice of physician	74%		--	
Changed physicians	49%		48%	
Physician primary health decision maker [1–10]	5.3 (1.2)		5.3 (1.2)	
Always better to seek medical help	44%		53%	
Foreign physician	9%		--	
Long waiting time with physician	16%		--	
Patients who had also accessed a non-primary care physician	33%		32%	
Outside interactions with physician	31%		--	
Number of visits to physician [1–4]	2.6 (1.2)		--	
**Encounter variables**	**Yes**	**No**	**Yes**	**No**
Major Surgery	10% (105/1045)	90%	10% (48/475)	90%
Minor surgery	18% (188/1045)	72%	16% (76/475)	84%
Prescribed medication that you thought might have serious side effect	15% (156/1045)	85%	16% (76/475)	84%
Overnight hospitalization	21% (220/1045)	79%	21% (99/475)	79%
Evaluated for possible or actual cancer	41% (428/1045)	59%	42% (199/475)	58%
Evaluated for another condition that you thought might be serious	40% (418/1045)	60%	43% (204/475)	57%
Referred to another doctor	57% (596/1045)	43%	57% (270/475)	43%

**Note: **Standard deviations () and ranges [] wherever applicable are indicated in parentheses and brackets.

Number of lifetime visits to the physician was categorized as follows: value of 1 denotes 2–5 visits, 2 denotes 6–10 visits, 3 denotes 11–20 visits, 4 denotes more than 21 visits.

* This column presents distribution of characteristics among persons who responded to items on physician trust.

** This column presents distribution of characteristics among persons who responded to items on general medical profession trust.

**Table 2:** Bivariate Spearman Rho Correlations between types of trust and patient-physician encounter variables in critical medical situations

**Dependent variable **⇒ **Independent variables **⇓	**Physician Trust (n = 1045)**	**Medical Profession Trust (n = 475)**
Major surgery	0.078*	0.012
Minor surgery	0.021	-0.00
Prescribed medication that you thought might have serious side effect	-0.12**	-0.1*
Overnight hospitalization	0.072*	0.13**
Evaluated for possible or actual cancer	0.048	-0.02
Evaluated for another condition that you thought might be serious	0.093**	-0.098*
Referred to another doctor	0.045	-0.013

**Notes: **Partial Spearman correlations are presented

* Significance at the 5% level

** Significance at 1% level for the test examining if partial Spearman ρ = 0

Dependent variable: Physician trust [10–50], and medical profession trust [11–55]. [] Range

**Table 3:** Partial correlation analysis results examining the relationship between patient-physician encounters and trust, adjusting for each other

**Dependent Variable **⇒	**Physician Trust** ** (n = 1045)**	**Dependent Variable **⇒	**Medical Profession Trust** ** (n = 475)**
**Independent Variables ** 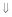		**Independent Variables ** 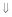	
**Encounter variables**		**Encounter variables**	
Major surgery (1 = yes, 0 = no)	0.057		
Prescribed medication that might have a serious side effect (1 = yes,0 = no)	-0.12**	Prescribed medication that might have a serious side effect (1 = yes,0 = no)	-0.03
Overnight hospitalization (1 = yes,0 = no)	-0.014	Overnight hospitalization (1 = yes,0 = no)	0.12**
Evaluated for another condition you thought to be serious (1 = yes,0 = no)	0.08**	Evaluated for another condition you thought to be serious (1 = yes,0 = no)	-0.079
**Other independent variables**		**Other independent variables**	
Satisfaction with health care (1 = yes,0 = no)	0.53**	Physician trust	0.21**
Poor physical health (1 = yes,0 = no)	-0.077*	Satisfaction with health care	-0.045**
Number of visits to physician	0.099**	Age	-0.12**
		Age-squared	0.13**
Foreign physician (1 = yes,0 = no)	-0.09**	Poor mental health (1 = yes,0 = no)	-0.084
Long waiting time with physician (1 = yes,0 = no)	-0.13**	Low income	0.016
Disputes with physician (1 = yes,0 = no)	-0.055	Changed physicians (1 = yes,0 = no)	-0.27**
Outside interactions with physicians (1 = yes,0 = no)	0.085**	Physician primary health decision maker (1 = yes,0 = no)	0.12**
Adequate choice in selecting physicians (1 = yes,0 = no)	0.12**	Always better to seek medical than self-treat (1 = yes,0 = no)	0.17**
Non-Physician is Primary care Provider (1 = yes,0 = no)	0.089**		

Notes: Partial Spearman correlations are presented

* Significance at 5% level

** Significance at 1% level for the test examining if partial Spearman ρ = 0

Dependent variable: Physician trust [10–50] and medical profession trust [11–55]. [] Range
